# Clinical study of ferredoxin-reductase-related mitochondriopathy: Genotype-phenotype correlation and proposal of ancestry-based carrier screening in the Mexican population

**DOI:** 10.1016/j.gimo.2023.100841

**Published:** 2023-11-11

**Authors:** Teresa Campbell, Jesse Slone, Hallie Metzger, Wensheng Liu, Stephanie Sacharow, Amy Yang, Mariya Moosajee, Chiara La Morgia, Valerio Carelli, Flavia Palombo, Matthew A. Lines, A. Micheil Innes, Rebecca J. Levy, Derek Neilson, Nicola Longo, Taosheng Huang

**Affiliations:** 1 Department of Pediatrics, Jacobs School of Medicine and Biomedical Sciences, University at Buffalo, Buffalo, NY; 2 Division of Genetics and Genomics, Boston Children’s Hospital, Boston, MA; 3 Harvard Medical School, Boston, MA; 4 Oregon Health and Science University, Portland, OR; 5 Institute of Ophthalmology, University College London, London, United Kingdom; 6 The Francis Crick Institute, London, United Kingdom; 7 IRCCS Istituto delle Scienze Neurologiche di Bologna, Programma di Neurogenetica, Bologna, BO, Italy; 8 Department of Biomedical and Neuromotor Sciences, University of Bologna, BO, Italy; 9 Department of Medical Genetics and Alberta Children's Hospital Research Institute, Cumming School of Medicine, University of Calgary, Calgary, Alberta, Canada; 10 Department of Neurology, Stanford University, Stanford, CA; 11 Division of Genetics and Metabolism, Department of Child Health, The University of Arizona College of Medicine, Phoenix, AZ; 12 Department of Pediatrics, University of Utah, Salt Lake City, UT

**Keywords:** FDXR, Ferredoxin reductase, Carrier screening, Mexican-Americans, Mitochondrial disease

## Abstract

**Purpose:**

Ferredoxin reductase (FDXR) is a flavoprotein that functions in both iron sulfur cluster biogenesis and steroid biosynthesis pathways in the mitochondria. Not surprisingly, loss of FDXR function causes severe mitochondrial diseases in humans. Although several FDXR-related mitochondriopathy (FRM) cohorts have been reported in the literature, further characterization of the natural history of FRM is warranted.

**Methods:**

To better understand the spectrum of FRM, a natural history study of FRM was performed. New cases were added to previously reported FRM cases for analysis (*n* = 62 cases).

**Results:**

Optic atrophy, movement disorder, and developmental delay were frequent findings. Mortality is high, with 18% of patients, often infants, passing from complications. Notably, 25% of cases were homozygous or compound heterozygous for the previously reported p.Arg386Trp “hotspot” variant. Of the obtained ancestry, all but 1 individual heterozygous for the p.Arg386Trp variant was Hispanic, with many reporting Mexican heritage. Utilizing recent large-scale genome sequencing surveys, the carrier frequency of the p.Arg386Trp variant was estimated as 1 of 185 in the Mexican population.

**Conclusion:**

Given the high mortality of FRM and carrier frequency of the common variant, consideration of a new approach for population carrier screening and development of therapeutics for affected individuals is needed.

## Introduction

Ferredoxin reductase (FDXR) is a flavoprotein localized on the inner mitochondrial membrane. It is responsible for transferring electrons acquired from nicotinamide adenine dinucleotide phosphate (NADPH) to the ferredoxin proteins. These downstream ferredoxin proteins, FDX1 (ferredoxin 1) and FDX2 (ferredoxin 2), have distinct roles within human tissues. FDX1 is primarily expressed in the adrenal gland where it transfers electrons to mitochondrial cytochromes P450 for steroid and vitamin D biogenesis. FDX2 is ubiquitously expressed and functions in the iron sulfur cluster (Fe-S) biosynthesis pathway in mitochondria.^1^ Fe-S are integral for normal cellular function and act as inorganic cofactors for numerous proteins, including those essential for energy metabolism and mitochondrial electron transport chain activity.^2^ Therefore, because FDXR initiates electron transfer for the activity of ferredoxins, it is required for proper steroid production, Fe-S homoeostasis, and subsequent energy metabolism.

FDXR is coded by the *FDXR* gene located on chromosome 17q25.1, which comprises 12 exons. Expression of the *FDXR* gene results in the transcription of several alternatively spliced *FDXR* messenger RNA (mRNA) transcripts. Although multiple mRNA transcripts have been identified, it is unknown if all isoforms are translated. It is speculated that only 2 gene isoforms are utilized for FDXR production.^3^ Structurally, FDXR contains binding domains for NADPH and flavin-adenine dinucleotide. Proper binding is essential for FDXR redox function.

Given the important role of FDXR in various tissues, it is of no surprise that pathogenic variants in the *FDXR* gene cause clinically severe disease. In 2017, our group was one of the first to report that biallelic variants in the *FDXR* gene cause mitochondriopathy in a large cohort.^4^ Since that time, our group and colleagues have described many additional cases of FDXR-related mitochondriopathy (FRM), for a total of 48 cases reported at the time of this manuscript’s submission.^5^, ^6^, ^7^, ^8^, ^9^, ^10^ Common findings include optic atrophy, visual dysfunction, hearing loss, movement disorder, and global developmental delay. Within our 2017 cohort we uncovered a “hotspot” variant, termed p.Arg392Trp, which was found in the homozygous or compound heterozygous state in multiple individuals with FRM. For the purpose of this article, we are using updated nomenclature and will refer to this variant as p.Arg386Trp for the entirety of this manuscript. Clinically, these individuals were severely affected, with functional studies showing significantly decreased FDXR activity, mitochondrial dysfunction, and mitochondrial iron overload.^11^ Strikingly, we uncovered a naturally occurring homozygous variant, p.Arg389Gln, in the B6; 129 S-Fdxr^m1J^ Otop3^m1J^/GrsrJ mouse strain (Jackson Laboratory, Stock #026096), which results in a substitution at the same amino acid position as the p.Arg386Trp human pathogenic variant. These mice presented with vision loss and an ataxic gait, confirming a deleterious effect of substitutions at this amino acid position.^4^ Interestingly, besides the p.Arg386Trp variant, Jurkute et al reported several unrelated individuals with FRM who were compound heterozygous for a p.Pro372His variant, suggesting the existence of other “hotspot” variants.^7^ Taken together, the current data suggest the existence of founder variants and/or adaptive advantages of harboring heterozygous *FDXR* gene variants. Given the number of individuals reported since initial discovery, we hypothesize that FRM is more prevalent in the general population than was previously appreciated.

Here, we present 14 newly identified individuals with FRM and summarize previous cohorts from the literature. We aim to better define the clinical spectrum and molecular genetics of FRM, including symptom onset, common features, and genotype-phenotype analysis.

## Materials and Methods

### Participant recruitment

“Natural History of *FDXR* Mutation-related Mitochondriopathy” was posted on clinicaltrials.gov and started recruiting participants on October 9, 2020 (NCT04580979). Individuals, legal guardians, or representative physicians contacted the research team via the website listing. Virtual interviews were conducted with each participant or their representative. Inclusion criteria included individuals with biallelic variants in the *FDXR* gene with phenotype suspicious for FRM. Individuals heterozygous for a *FDXR* variant or individuals without known *FDXR* gene variants were not included in the study. Participants were asked to complete a questionnaire describing clinical symptoms (Supplemental Figure 1). Additionally, genetic test reports and medical records were requested for review. Variants were converted to NM_024417.5 nomenclature. Selected participants provided a skin biopsy for functional analysis. Signed informed consent was provided by all participants or their legal guardians.

### Fibroblast generation

Briefly, a skin biopsy (3 mm diameter) was collected from the upper shoulder of each participant, dissected into small pieces, incubated in 0.7% collagenase (type B) for 1.5 to 2.5 hours at 37 °C, and plated into 1 to 2 T25 flasks containing AmnioMAX™ C-100 Complete Medium (Thermo Fisher Scientific). Fibroblast cultures were subsequently maintained in Dulbecco’s Modified Eagle Medium (Thermo Fisher Scientific), supplemented with 10% Fetal Bovine Serum (Thermo Fisher Scientific). Normal human dermal fibroblasts were used as controls for all fibroblast studies.

### Reverse transcriptase polymerase chain reaction (RT-PCR)

Total cellular RNA was isolated using TRIzol Reagent (Thermo Fisher Scientific), according to the manufacturer’s instructions. RT-PCR reactions were set up using the Invitrogen SuperScript III One-Step RT-PCR System with Platinum Taq High-Fidelity DNA Polymerase (Thermofisher Scientific), according to the manufacturer’s instructions. The following primers were used: “Fdxr cds 193F” - GTGGACATCTACGAGAAACAG, and “Fdxr cds 800R” - CTTGATCTTGTCCTGGAGAC. Optimal amplification was found using an annealing temperature of 65.1 °C. To determine the exact sequence of the splice isoform present in participant 14, the band containing the alternate splice form was excised with a razor and gel purified using the QIAquick Gel Extraction Kit (Qiagen). The purified DNA was then Sanger sequenced with primer “Fdxr cds 193F” - GTGGACATCTACGAGAAACAG.

### Western blot

Western blot analysis was performed as previously described.^12^ Rabbit anti-FDXR antibody (ab204310) (Abcam) was applied at 1:1000 dilution. The antibody was raised against a recombinant protein fragment containing amino acids 1 through 150 of human FDXR. After stripping the membrane, it was re-probed with mouse anti-beta-actin antibody (NP600-501, AC-15) (Novus Biologicals, Englewood) at 1:1000 dilution as a loading control. The relative strength of each band was quantified using Image Lab Software from Bio-Rad (Bio-Rad).

### Calculation of carrier frequency

Public genome databases, The Genome Aggregation Database (gnomAD) (https://gnomad.broadinstitute.org/) and Mexico City Prospective Study Variant Browser (MCPS) (https://rgc-mcps.regeneron.com/), were utilized. Briefly, gnomAD includes 125,748 exome sequences and 15,708 genome sequences obtained from various international genetic studies.^13^ MCPS includes exome data of 150,000 adults and genome data of 10,000 individuals from Mexico City and surrounding areas.^14^ The Regeneron Genetics Center, University of Oxford, Universidad Nacional Autónoma de México, National Institute of Genomic Medicine in Mexico, Abbvie Inc., and AstraZeneca UK Limited (collectively, the “Collaborators”) bear no responsibility for the analyses or interpretations of the data presented here. Any opinions, insights, or conclusions presented herein are those of the authors of this paper. The “hotspot” *FDXR* c.1156C>T, p.Arg386Trp variant was queried, and allele frequency was notated. The Hardy-Weinberg equation (p^2^ + 2pq + q^2^ = 1) was used to estimate carrier frequency (2 pq) and frequency of affected individuals (q^2^). Prevalence was calculated for homozygotes in the Mexican population using (q^2^) (total population), as reported on the Mexico 2020 Census data obtained from https://inegi.org.mx.

## Results

### Clinical findings in new individuals with FRM

The research team was contacted 23 times by individuals, or their representative health care provider, interested in participating in the study. Of those, 21 had biallelic variants in the *FDXR* gene, and a suspected diagnosis of FRM, meeting inclusion criteria for the study. Fourteen participants were interviewed and consented to be included in this study, the remaining contacts were either lost to follow-up or passed away before consenting to the study (Table 1). The majority of participants were from North America, with 1 participant each recruited from Australia, United Kingdom, Italy, and Spain. Ten of the participants were females and 4 of the participants were males. Ages ranged from 1 to 30 years. Nine of the 14 participants were under the age of 5, showing a bias toward early-onset disease in this cohort. Optic atrophy was reported in 13 of the participants. Similarly, 13 participants presented with a movement disorder, mainly ataxia. Eleven of the 14 participants had developmental delay ranging from mild to severe. The severity of delays frequently correlated with an earlier age of onset. In the more severe cases, many parents reported regression of development. The most common trigger for regression was viral illness. For participants with early-onset FRM, illness frequently resulted in hospitalizations and, for a single participant, early death. Interestingly, the oldest participant in our cohort (participant 7) reported successful treatment with corticosteroids during periods of regression. However, this approach was no longer effective as he became older. Participants in our cohort over the age of 10 had milder symptoms, more commonly presenting with a combination of optic atrophy and hearing loss. Moreover, these older participants did not present with intellectual disability or have a history of learning delay. Six of the 14 participants in the cohort were homozygous or compound heterozygous for the common p.Arg386Trp variant. Eight of 9 heterozygous parents reported Mexican heritage, with the remaining heterozygous parent from Spain. The majority of patients obtained a molecular diagnosis via exome sequencing, with a few patients obtaining a molecular diagnosis through panel testing (ie, neurodevelopmental panel, mitochondrial disease panel, and retinal dystrophy panel).

**Table 1:** Newly reported participant phenotypes and genotypes with FDXR-related mitochondrial diseases

Participant ID	1	2	3	4	5	6	7	8	9	10	11	12	13	14
Sex	F	M	F	F	F	F	M	M	F	F	F	F	F	M
FDXR variants^a^	c.1156C>T, p.Arg386Trp	c.1208C>T, p.Pro403Leu c.1156C>T, p.Arg386Trp	c.925C>T, p.Arg309Ter c.1208C>T, p.Pro403Leu	c.925C>T, p.Arg309Ter c.1208C>T, p.Pro403Leu	c.1189G>A, p.Gly397Ser c.1003-6C>G, p.Gly335Alafs∗8	c.916C>T, p.Arg306Cys	c.1102G>A, p.Asp368Asn c.1A>G, p.Met1Val	c.1156C>T, p.Arg386Trp	c.1156C>T, p.Arg386Trp c.270+5 G>T, p.?	c.1156C>T, p.Arg386Trp	c.463C>T, p.Arg115Trp c.802+3G>C, p.?	c.980G>A, p.Arg327His c.1382_1392del insATCC, p.Leu461fs	c.1156C>T, p.Arg386Trp c.1189G>A, p.Gly397Ser	c.754C>T, p.Gln252∗ c.339G>A p.?
	homozygous	in *trans*	in *trans*	in *trans*	in *trans*	homozygous	in *trans*	homozygous	in *trans*	homozygous	in *trans*	in *trans*	in *trans*	in *trans*
Age at consent	5 y	2 y	8 y	3 y	11 y	12 y	30 y	12 mo	14 mo	2 y	6 y	26 y	21 mo	4 y
Age of onset	4 mo	5 mo	9 mo	9 mo	18 mo	7 y	2.5 y	3 mo	4 mo	4 mo	9 mo	21 y	3 mo	6 mo
Optic atrophy	Yes	No	Yes	Yes	Yes	Yes	Yes	Yes	Yes	Yes	Yes	Yes	Yes	Yes
Vision loss	Yes	Yes	Yes	Yes	Yes	Yes	Yes	Yes	Yes	Yes	Yes	Yes	Yes	Yes
Strabismus	Yes	No	Yes	No	Yes	Yes	Yes	No	Yes	No	Yes	No	No	No
Nystagmus	No	Yes	Yes	Yes	Yes	No	Yes	Yes	No	Yes	No	No	No	Yes
Retinopathy	No	No	Yes	No	No	No	No	No	No	No	Yes	Yes	No	No
Hearing loss	No	Yes	No	No	No	Yes	Yes	No	No	Yes	No	No	Yes	No
Developmental delay	Yes	Yes	Yes	Yes	Yes	No	No	Yes	Yes	Yes	Yes	No	Yes	Yes
Regression	Yes	Yes	Yes	Yes	Yes	No	Yes	Yes	Yes	Yes	No	No	Yes	No
Hypotonia	Yes	Yes	Yes	Yes	Yes	No	No	Yes	Yes	Yes	Yes	No	Yes	Yes
Movement Disorder	Yes	Yes	Yes	Yes	Yes	No	Yes	Yes	Yes	Yes	Yes	Yes	Yes	Yes
Spasticity	Yes	No	Yes	No	No	No	No	No	No	Yes	No	No	Yes	No
Seizures	No	No	No	No	Yes	No	No	No	No	Yes	No	No	Yes	No
Other brain MRI findings	None reported	Cerebellar degeneration	Swelling of cerebellum	None reported	Progressive cerebellar atrophy	None reported	Cerebellar degeneration	Delayed myelination, nonspecific prominence of the ventricles and subarachnoid spaces	None reported	Midline anomalies: absence of the septum pellucidum, reduced optic apparatus, severe hypoplasia of olfactory apparatus	None reported	Thinned optic chiasm	Not provided	None reported
Other	Iron staining on teeth	None reported	None Reported	None reported	Hypermobility, headaches	None reported	Regressions treated with prednisone in childhood, psychiatric disturbances	None reported	Hypertension	History of hyperammonemia, hypertension	None reported	Cataracts, Marfanoid habitus, Hypermobility, spinal cord atrophy	None reported	Microcephaly, complex I, IV deficiency

*F,* female; *M,* male; *mo*, month; *MRI,* magnetic resonance imagining; *y*, years.

a Listed in NM_024417.5.

### Review of the clinical findings of all reported FRM individuals

**Figure 1:**
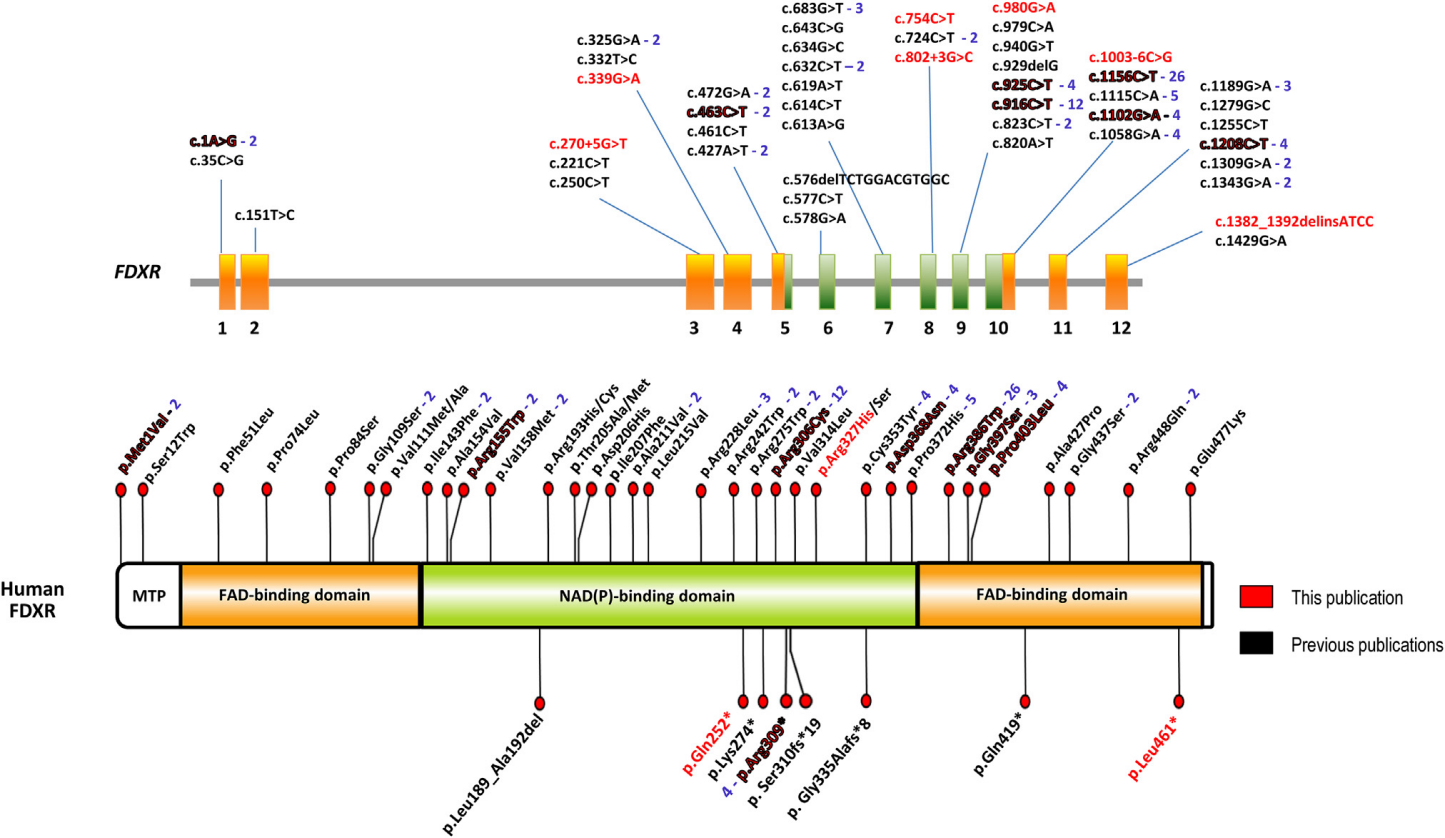
**Location of participant variants in cDNA (upper) and protein (lower) sequence.** Splice variants not predicted on protein sequence. Blue numbers (- #) represent the number of times this allele was reported in the literature; variants are using nomenclature NM_024417.5. Domain labeling is based on previously published crystallography analysis of the *Bos taurus* ortholog of FDXR.^15^ FDXR, ferredoxin reductase; MTP, mitochondrial transit sequence.

Review of the literature disclosed 48 previously published cases of FRM at the time of manuscript submission. In addition to the new cohort, 62 individuals in total were included for analysis (Table 2). Similar to the findings in the new cohort, optic atrophy was the most common feature (92%), with movement disorder (84%) being next. Phenotype review can categorize individuals with FRM into 2 generalized cohorts, early-onset (symptom onset before 2 years of age) and late-onset disease. Of the early-onset cases, the first presenting symptom is typically ocular, with nystagmus, strabismus, and/or abnormal gaze presenting within the first 1 to 2 years of life. All individuals appear typically developing at birth. Ocular findings usually progressed to optic atrophy and severe vision loss. Global developmental delay was ubiquitous in these individuals. Unfortunately, all of these individuals had regression within the first 1 to 2 years of life, frequently following a viral illness. To our knowledge, 9 individuals from the literature and 1 from our current study had passed from medical complications related to early-onset FRM, suggesting a high mortality rate (18%). Among late-onset individuals, ocular findings, hearing loss, and variable late-onset ataxia were common. Almost all present with optic atrophy and progressive vision loss. Although regression was reported in several of these individuals, critical hospitalizations were less likely to occur. With regard to genotype, reported variants were prevalent throughout the *FDXR* coding regions. Exons 7 through 10, which code for sections of the NADPH-binding domain, harbored the highest frequency of variants (Figure 1). Domain labeling is based on previously published crystallography analysis of the *Bos taurus* ortholog of FDXR.^15^ Fifteen individuals were homozygous or compound heterozygous for the p.Arg386Trp variant, for a combined total of ∼25% of the total cohort. This variant was frequently identified in early-onset FRM. Besides the p.Arg386Trp variant, 7 individuals were homozygous or compound heterozygous for the p.Arg306Cys variant; however, 4 of these individuals were from the same consanguineous family. The phenotype of individuals homozygous for p.Arg306Cys was optic atrophy and auditory neuropathy.^8^ Similarly, individuals with the p.Pro372His common variant presented at later ages with optic atrophy, hearing loss, and progressive ataxia. The most unique phenotype was reported in our 2018 article, with “case #2” presenting with characteristic findings of early-onset FRM in addition to a phenotype suggestive of a disorder of sexual development.^6^ Overall, the spectrum of FRM can vary; however, optic atrophy is present in nearly all individuals with FRM.

**Table 2:** Summary of clinical findings in participants with FDXR-related mitochondrial diseases

Clinical Findings	Previous Work^a^	Current Study	Total^b^
Optic atrophy	44	13	57/62 = 92%
Strabismus	5	7	12/56 = 21%
Nystagmus	11	8	19/56 = 34%
Retinopathy	13	3	16/56 = 29%
Hearing loss	28	5	33/62 = 53%
Developmental delay	19	11	30/57 = 53%
Regression with illness	19	10	29/57 = 50%
Hypotonia	24	11	35/56 = 63%
Movement disorder	35	13	48/57 = 84%
Spasticity	13	4	17/56 = 30%
Seizures	5	3	8/57 = 14%
Microcephaly	7	1	8/39 = 21%
Homozygous or compound heterozygous for p.Arg386Trp	8	6	14/57 = 25%
Death^c^	9	1	10/57 = 18%
Other	Hypertension, respiratory distress/failure, sepsis, ambiguous genitalia, and hypermobility		

% rounded to nearest integer.

a 4-10.

b If addressed by reporting publication.

c At the time of publication.

### Splice-site analysis

**Figure 2:**
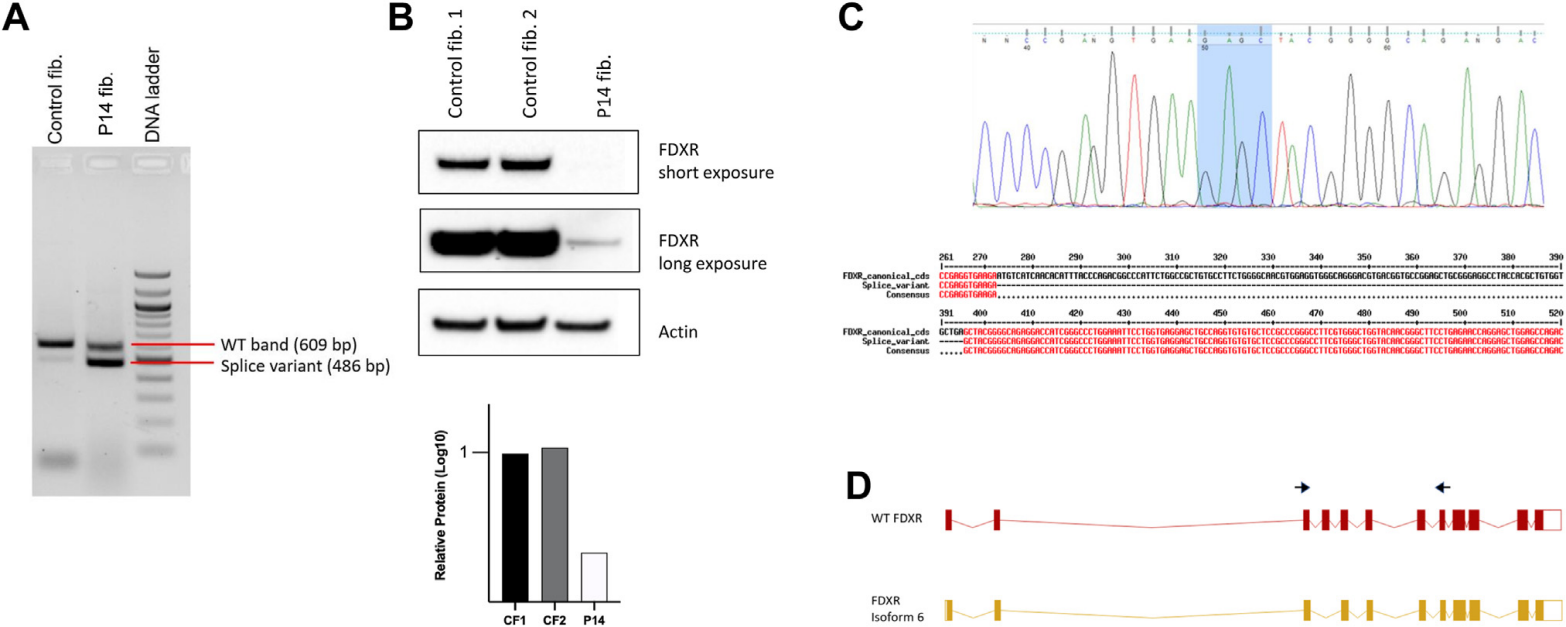
**Splice site analysis for participant 14.** A. RT-PCR of exons 3-7 of FDXR transcript in control and participant 14 (P14) fibroblasts (fib.). B. Western blot and the relative FDXR protein in participant fibroblasts. C. Sanger sequencing and aligned sequences of splice site junction. D. Schematic of WT and isoform 6 FDXR transcripts. Normal human dermal fibroblasts were used as controls for all fibroblast studies. FDXR, ferredoxin reductase; WT, wild type.

Participant 14 was very unique. He was self-referred to our study after his parents reached out to our group following our first publication. He carried a paternally inherited, likely pathogenic, stop-gain variant (c.754C>T, p.Gln252∗) and a maternally inherited, synonymous, variant of uncertain significance (c.339G>A, chr17:72,862,622) in the *FDXR* gene. In silico predictions suggested a possible splice-site alteration. To directly evaluate the impact of the c.339G>A variant on FDXR function, cultured fibroblasts were utilized for RNA and protein studies. RNA analysis identified 2 alternatively spliced transcripts: a weaker 609 bp band, consistent with the wild-type *FDXR* mRNA transcript and a shorter 486 bp band suggestive of an alternatively spliced transcript (Figure 2A). Western blot showed that participant 14 had little to no FDXR protein in fibroblast sample (Figure 2B). To confirm these findings, Sanger sequencing of the resulting cDNA at the splice-site junction confirmed the precise location of the novel splice site. To our surprise, alignment of this alternatively spliced sequence with the canonical transcript revealed an in-frame deletion (Figure 2C). Further analysis determined that this alternative transcript spliced out all of exon 4, consistent with isoform 6 of the FDXR protein (NM_001258015.3 → NP_001244944.1). These data, together with the results of the western blot, suggest that this isoform does not result in a functional protein (Figure 2D). Because c.339G>A is in trans with a nonsense variant (c.754C>T, p.Gln252∗), this analysis confirmed that the c.339G>A variant results in an alternatively spliced transcript, which is either not translated or possibly leads to an unstable protein product.

### Estimated carrier frequency of common variant p.Arg386Trp

**Figure 3:**
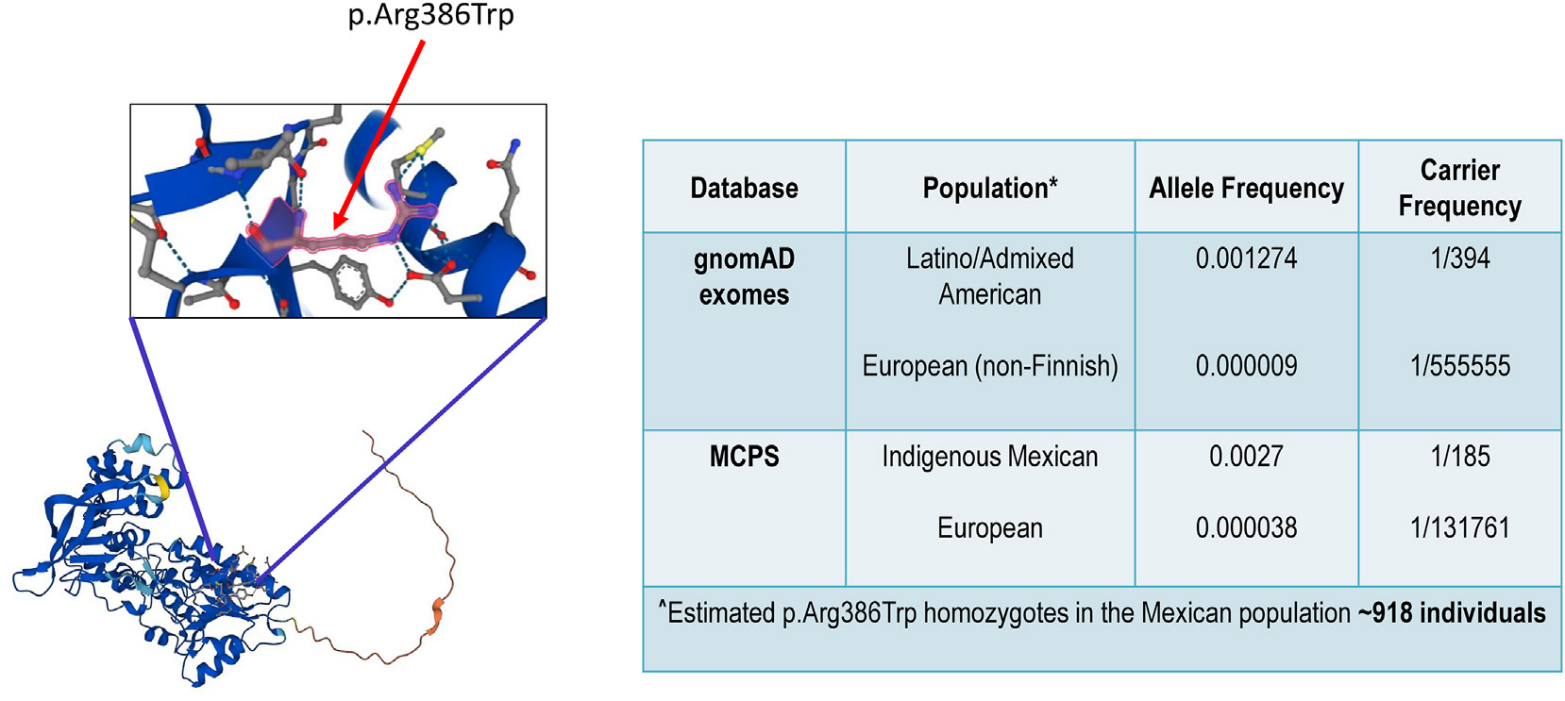
**Location and carrier frequency of p.Arg386Trp in various genome databases.** Protein structure obtained from AlphaFold (https://alphafold.ebi.ac.uk/entry/Q6GSK2). gnomAD, The Genome Aggregation Database (https://gnomad.broadinstitute.org/); MCPS, Mexico City Prospective Study Variant Browser (https://rgc-mcps.regeneron.com/) ∗Populations with allele frequency of zero not included in table. ˆEstimated from 2020 Mexico population census reported on https://inegi.org.mx.

Within our newly identified cohort, 6 of the 14 participants (∼42%) recruited were homozygous or compound heterozygous for the p.Arg386Trp variant. Of those participants, 5 of 6 cases reported Mexican heritage in 1 or both parents. The remaining participant was from a Spanish family. Of previously reported individuals, 9 carried the p.Arg386Trp variant.^4^^,^^5^ Of those families who disclosed ethnicity, all identified as Hispanic. Given this unusual finding, we hypothesized that our previously identified “hotspot” variant is prevalent in Mexican Americans and/or Mexicans. Review of this variant in the public genome databases MCPS and gnomAD disclosed an allele frequency of 0.0027 and 0.001274, respectively, representing Indigenous Mexican and Latino/Admixed American populations. Allele frequency was null in several ethnic populations. The p.Arg386Trp variant was detected at low frequency in Europeans in gnomAD exomes (frequency 0.000009), African/African Americans in gnomAD genomes (0.000024), and Europeans in the MCPS (0.000038). Calculations using Hardy-Weinberg equation (p^2^ + 2pq + q^2^ = 1) determined an estimated carrier frequency of 1 in 185 and 1 in 394 in Indigenous Mexicans and Hispanic populations, respectively. Assuming q^2^ = 0.00000729, for the estimated population of Mexico from 2020 census data (*n* = 126,014,024), we project that 918 individuals are homozygous for the deleterious p.Arg386Trp variant (Figure 3). We assumed no selection against q^2^ as, currently, we have no evidence to suggest that p.Arg386Trp is embryonic lethal in the homozygous state. Parents who are heterozygous for the p.Arg386Trp pathogenic variant do not report a history of recurrent miscarriage. Additionally, individuals homozygous for the p.Arg386Trp pathogenic variant are typically normal appearing at birth and progressively show signs of FRM. This estimation is likely a vast underestimate because this does not account for other pathogenic variants in the *FDXR* gene that contribute to disease (Figure 1). Following up on this analysis, protein structure and location of the p.Arg386Trp variant was then visualized using Alphafold software, showing that the p.Arg386Trp variant is located within the flavin-adenine dinucleotide/NAD(P) binding domain (https://alphafold.ebi.ac.uk/entry/Q6GSK2).^16^ Because we had previously shown that FDXR protein is significantly reduced in individuals homozygous for the p.Arg386Trp variant, we hypothesize the p.Arg386Trp variant disrupts FDXR protein stability (Figure 3).

## Discussion

We report 14 newly identified individuals with FRM uncovering several new aspects of this disease and providing a comprehensive and updated review of FRM. From this assessment, a clear differentiation between early-onset and late-onset disease is emerging.

Early-onset FRM is a devastating condition. Unfortunately, multiple early-onset participants died in childhood, often suddenly, after a brief period of deterioration. Based upon functional studies, the genotypes of these participants suggest severe loss of FDXR function. Notably, the common p.Arg386Trp variant in the homozygous state, or in combination with a null allele, is catastrophic, causing severe early-onset disease. Concerning the pathophysiology, one mechanism is mitochondrial dysfunction caused by iron overload, dysfunctional electron transport chain, and adenosine triphosphate depletion. This dysfunction is then exacerbated during a period of illness that requires increased energy production. However, given the role of FDXR in the biogenesis of steroid hormones in the adrenal gland, a concerning aspect of early-onset disease may be undiagnosed adrenal insufficiency. In this study, participants 10 and 12 were reported to have uncontrolled hypertension, which can be reported in disorders of steroidogenesis, such as 11-beta-hydroxylase deficiency, caused by biallelic variants in the *CYP11B1* gene, a downstream target of FDXR in the adrenal gland. Additionally, participant 7 reported successful treatment with corticosteroids during periods of regression during childhood and adolescence. More convincingly, in our 2018 publication, “case #2” presented with ambiguous genitalia, a common finding in disorders of sexual development caused by primary deficiencies in hormone production.^6^^,^^17^ Taken together, these clinical findings cannot be explained as resulting from iron overload and adenosine triphosphate depletion in the mitochondria. Because lifesaving interventions can be implemented during adrenal crisis, this possible aspect of early-onset FDXR disease warrants attention and future evaluation in individuals with FRM.

Although the majority of our cohort presented with early-onset FRM, later-onset FRM was also reported. Unpublished data from our group suggest that *FDXR* variants causative of later-onset FRM allow for residual function of the FDXR protein. The case of participant 7 may also be an instructive example. Although the onset of disease in this individual began as early as 2.5 year of age, this individual has reached a much more advanced age that the more severe early-onset FRM cases (being consented at the age of 30 years old) and has managed to avoid many of the more severe neurological presentations, such as hypotonia and developmental delay. A look at the specific variants present in this individual suggests several possibilities for residual FDXR function. For example, it is possible that the c.1A>G variant (which would be expected to eliminate the canonical FDXR start codon) is compensated by the use of an alternate start site. It is also possible that the aspartate to asparagine substitution created by the other variant present in this individual (c.1102G>A, p.Asp368Asn) causes only a minor disruption in FDXR function because of the similarity of those 2 amino acids. Regardless, considering the delayed onset and slower progression of cases such as this, iron accumulation leading to mitochondrial dysfunction is a more logical hypothesis for the pathophysiology of later-onset disease. Because optic atrophy and retinal degeneration cannot be reversed after retinal ganglion cell loss, research into preventative therapies targeting iron accumulation and/or mitochondrial dysfunction should be explored. Moreover, *FDXR* gene analysis should be considered for patients with unexplained adult-onset optic atrophy and/or retinopathy, especially in the context of additional findings such as hearing loss or movement disorder.

Because FRM is a relatively newly described condition, it is not surprising that many reported clinical variants are classified as variants of uncertain significance. Although data sharing of variant classification in combination with phenotyping is important for prospective reclassification, functional experimentation can clarify variant pathogenicity. Functional analysis using fibroblasts from participant 14 demonstrated that the majority of mRNA transcript in the cells was from the c.339G>A allele. This mRNA was alternatively spliced and significantly decreased the amount of FDXR protein in the cell. Moreover, this alternatively spliced mRNA had 100% sequence identity with FDXR transcript variant 6, suggesting that the c.339G>A variant is influencing the choice of splice site such that isoform 6 is being produced at a much higher frequency than would normally be the case. The transcript for FDXR isoform 6 has been shown to be detectable at low levels in normal human blood samples and shows a modest level of upregulation in response to ionizing radiation (although to a far lesser extent than the other FDXR isoforms).^18^ However, our western blot analysis indicates that this transcript is either not efficiently translated into a functional FDXR protein, or the translated protein is unstable, resulting in degradation. We thus question the role of these alternatively spliced mRNAs and whether they contribute to overall FDXR function. Another hypothesis is that alternative transcripts are utilized in different tissues, affecting tissue-specific protein function. Regardless, clarification of this variant pathogenicity was vital for molecular diagnosis for the participant. Moreover, this information was used for reproductive planning for this family. Although our current sequencing technologies are increasing in sensitivity, deep intronic or splice-site variants are often overlooked. Our data highlight the need for functional analysis and characterization of participant samples when the phenotype is consistent and only a single pathogenic variant is initially identified.

One of the most important aspects of our study is the estimated carrier frequency of the p.Arg386Trp variant in individuals with Mexican heritage. Ancestry-based carrier screening has been practiced in the United States for several decades.^19^ Depending on the center, couples are offered standard population and ancestry-based carrier screening, with the option of expanded carrier screening. Diseases of significance include cystic fibrosis, hemoglobinopathies, Fragile-X, Tay-Sachs disease, and spinal muscular atrophy. Several professional organizations have published carrier screening guidelines for specific ethnic groups.^20^ Criteria for the inclusion of a condition suitable for population screening has been published by a variety of organizations, including the American College of Obstetricians and Gynecologists, the American College of Medical Genetics and Genomics, and National Society of Genetic Counselors. To summarize, these criteria state that the disease should have (1) a significant impact on the quality of life of the affected individual, (2) a high carrier frequency in the screened population, (3) a valid methodology for screening, (4) a well-defined genotype-phenotype correlation, and (5) prenatal diagnosis and intervention are available reproductive options.^21^ FRM caused by the p.Arg386Trp variant meets all the aforementioned criteria. Given the severity of disease for children who are homozygous or compound heterozygous for the p.Arg386Trp, we strongly advocate for FRM carrier screening to be offered to individuals of Mexican heritage as part of standardized preconception counseling and future family planning.

Since our initial report defining FRM, we have been struck by the number of confirmed individuals reported in the literature. Moreover, during the 28 months of our study being posted on clinicaltrials.gov, at the time of this publication, we have been contacted 21 times by physicians or families disclosing newly confirmed cases of FRM. Taking this into account, in combination with the estimated carrier frequency of p.Arg386Trp, we foresee a large cohort of undiagnosed individuals within North America. This heightens the urgent need to develop effective therapies for this population. We have previously shown that adeno-associated virus gene therapy was effective in Fdxr deficient mice.^22^ Importantly, although early-onset is reported, FRM has a postnatal onset, making gene therapy feasible for this population. Gene therapy is a promising future direction to help aid in the quality of life and hopefully to decrease mortality for these individuals.

Taken together, this work broadens the spectrum of FRM, showing a differentiation between early- and late-onset disease. In addition, this study presents functional studies of previously reported variants of uncertain significance, further implicating the impact of splice-site variants. Lastly and most importantly, characterization of the previously reported “hotspot” variant c.1156C>T, p.Arg386Trp estimates a striking carrier frequency in individuals from Mexico or with Mexican heritage, raising the question of whether FDXR should be recommended for ancestry-based carrier screening in this population.

## Data Availability

Deidentified data and materials are available upon request.

## Conflict of Interest

The authors declare no conflicts of interest

## Additional Information

Supplementary File Sample copy of the patient medical history questionnaire for “Natural History Study of FDXR Mutation-related Mitochondriopathy.”
