# Supplementary material for: Clinical study of ferredoxin-reductase-related mitochondriopathy: Genotype-phenotype correlation and proposal of ancestry-based carrier screening in the Mexican population
Source: Genet Med Open. 2023 Nov 11;2:100841. doi: 10.1016/j.gimo.2023.100841 (PMC11613914; doi:10.1016/j.gimo.2023.100841)
Supplement: Supplementary File — Sample copy of the patient medical history questionnaire for “Natural History Study of FDXR Mutation-related Mitochondriopathy.” [file mmc1.pdf]

Medical History Questionnaire for "Natural History Study of FDXR Mutation-related Mitochondriopathy"

The following is a sample of the medical history that will be requested should you agree to participate. Please do not forward any information until you have contacted the study officials and provided formal consent to participate in this study. For more information, please contact the primary study official using the contact information below:

Study Contact: Taosheng Huang, MD, Ph.D.
Address: 1001 Main Street, Room 5116
Buffalo, NY 14203
Email: thuang29@buffalo.edu
Phone: 716-323-0041
Fax: 716-323-0292

I. PATIENT INFORMATION

Date of visit: ___________________________

Patient Name:______________________________

Gender: _______________________________

Age: _________________

Date of birth: _______________

Mother's Name (if patient is under 18): ____________________________

Father's Name (if patient is under 18): _____________________________

II. STUDY-RELATED MEDICAL HISTORY

Condition	YES	NO	Age of symptoms	Anything else you want to tell us
**Known mutations in *FDXR***				(please provide genotype if known or attach a report)
**Any family history of illness**				Please attach a family tree
**Complications of pregnancy**				
**Premature birth**				

**Complications with birth**				
**Developmental delay**				
**Developmental regression**				
**Abnormal size of brain**				
**Movement disorders (ataxia, dystonia, etc.)**				
**Seizures**				
**Optic atrophy in eye exam**				
**Vision loss**				
**Other vision problems (color, eye movement)**				
**Hypotonia (muscle weakness or lack of tone)**				
**Electromyogram (EMG)**				Please attach the report
**Muscle biopsy**				
**Spasticity (muscle stiffness or tightness)**				
**Brain MRI**				Please attach the report
**Electroencephalogram (EEG)**				Please attach the report

III. GENERAL MEDICAL HISTORY

Condition	YES	NO	Age of symptoms	Anything else you want to tell us

**Abnormality in Head, Ears, Eyes, Nose, and Throat)**				
**Headache**				
**Cardiovascular**				
Abnormal Blood Pressure				
Irregular heart beat				
Stroke				
Other				
**Respiratory**				
Asthma				
Difficult to breathe				
Pneumonia				
Pulmonary Disease				
Other				
**Endocrine & Metabolic**				
Diabetes Mellitus				
Thyroid Disorder				
Pancreatic disorder				
Short statue				
Other				
Immune System				
Infection				
Other				
**Hematology & Lymphatic**				
Thrombocytopenia				
Anemia				
Other				
**Dermatological**				
Rashes				

Lesions				
Musculoskeletal				
Pain				
Arthritis				
Weakness				
Other				
Gastrointestinal/Digestive				
Reflex				
Digestion issue				
Constipation				
Diarrhea				
Other				
**Genitourinary**				
Kidney stones				
Renal Insufficiency				
Neurologic/ Psychiatric				
Depression				
Anxiety				
Other neurologic				
**Procedures/Surgical History**				
**Other Medical History**				
